# Induced Pluripotent Stem Cell as a New Source for Cancer Immunotherapy

**DOI:** 10.1155/2016/3451807

**Published:** 2016-02-25

**Authors:** Farzaneh Rami, Halimeh Mollainezhad, Mansoor Salehi

**Affiliations:** ^1^Department of Genetics and Molecular Biology, School of Medicine, Isfahan University of Medical Sciences, Isfahan 81746-73461, Iran; ^2^Department of Immunology, School of Medicine, Isfahan University of Medical Sciences, Isfahan 81746-73461, Iran

## Abstract

The immune system consists of cells, proteins, and other molecules that beside each other have a protective function for the host against foreign pathogens. One of the most essential features of the immune system is distinguishability between self- and non-self-cells. This function has an important role in limiting development and progression of cancer cells. In this case, the immune system can detect tumor cell as a foreign pathogen; so, it can be effective in elimination of tumors in their early phases of development. This ability of the immune system resulted in the development of a novel therapeutic field for cancer treatment using host immune components which is called cancer immunotherapy. The main purpose of cancer immunotherapy is stimulation of a strong immune response against the tumor cells that can result from expressing either the immune activator cytokines in the tumor area or gene-modified immune cells. Because of the problems of culturing and manipulating immune cells* ex vivo*, in recent years, embryonic stem cell (ESC) and induced pluripotent stem cell (iPSC) have been used as new sources for generation of modified immune stimulatory cells. In this paper, we reviewed some of the progressions in iPSC technology for cancer immunotherapy.

## 1. Introduction

The immune system consists of cells, proteins, and other molecules that beside each other have a protective function for the host against foreign pathogens. This system has two major types called innate and adaptive immunity. The innate immune system acts as the first response against a pathogen that is a rapid and nonspecific response and has the ability to activate adaptive immune response [1, 2]. Some of the essential components of this system are macrophages, NK cells, dendritic cells (DCs), mast cells, neutrophils, and complement proteins. The adaptive immune system consists of B- and T-cells that can recognize antigens in a highly specific manner. Antibodies released by plasma cells make up the noncellular portion of the adaptive immune system [2].

One of the most essential features of the immune system is distinguishability between self- and non-self-cells. This function especially has an important role in limiting development and progression of cancer cells. In this case, the immune system can detect tumor cell as a foreign pathogen; so, it can be effective in inhibition and elimination of tumors in their early phases of development [1]. Tumor cells have some mechanisms for escaping from an immune response, for example, reduction or absence of surface MHCI expression in tumor cells [3], defective or altered apoptotic signaling pathways [4], reduced expression of adhesion molecules in blood vessels of tumor mass for reducing the ability of immune cells to migrate into tumor area [5], and secretion of immune suppressor cytokines [6]. The detection of these escaping mechanisms and the different responses of the immune system to cancer cells resulted in the development and progression of a novel therapeutic field for cancer treatment using immune components which is called cancer immunotherapy [5, 7].

The main purpose of cancer immunotherapy is stimulation of a strong immune response against the tumor cells using the components of the host immune system. This strong response can result from expressing either the immune activator cytokines and antibodies in the tumor area or gene-modified immune cells [8, 9]. The immunological checkpoint blockade is also a new strategy for cancer immunotherapy whose main purpose is enhancing tumor-specific activity of immune system. The central components for immunological checkpoint blockade strategy are immunoglobulin superfamily and cell surface receptors such as CTLA4 that can act either as activators for initiation of an immune costimulatory signal or as inhibitors for initiation of an immune coinhibitory signal in the targeted tumor cells. This strategy can be used alone or in combination with other cancer immunotherapy methods [10].

As declared, the immune cells are the most important and central components for most of the immunotherapy methods; because of the problems of culturing and manipulating immune cells* ex vivo*, in recent years, embryonic stem cell (ESC) has been used as a new source for generation of modified immune stimulatory cells [11]. The ESC is a pluripotent cell with the ability to differentiate to most tissue cell types including most of the immune system cells [11, 12].

**Figure 1:**
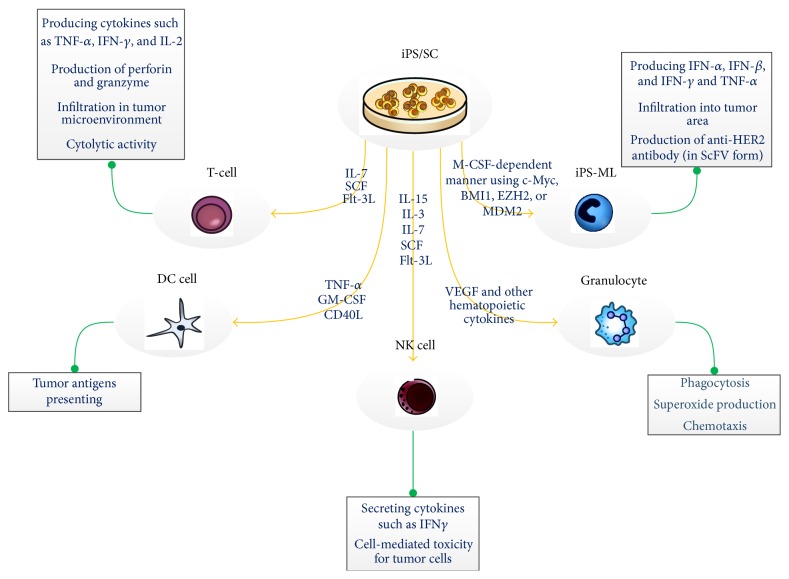
iPSC can differentiate into the immune system cells using some factors. These differentiated immune cells were indicated to have the ability to activate an immune response in different manner. Some of the factors for generating immune cells from iPSC and the function related to these cells are summarized in this schematic figure [14–18].

iPSC (induced pluripotent stem cell) is one of the other types of pluripotent cells with the same properties of ESC which made it a suitable choice for generation of immune cells. iPSC can be generated from a patient's somatic cells, such as blood cells and fibroblasts; then, it has the same genetic and histological structure of the patients cells. Using this kind of cell, it might be possible to generate a personal immune cell with antitumor activity that can be effective in personal cancer treatment. Moreover, it does not have the ethical concerns related to ESC and the problem of undesired immune reaction against a foreign tissue [13].  Figure 1 summarized some of the factors for generating immune cells from iPSC and the function related to these generated cells.

In this paper, we reviewed some of the progressions in iPSC technology for cancer immunotherapy.

## 2. iPSC Applications in Stimulation of Antitumor Immune Response

Today, using the immune system for cancer therapy has progressed in different areas such as cancer vaccines, T- and NK cell therapy, antitumor antibodies, immune regulatory cytokines, and DC cancer therapy [5, 7]. These therapeutic methods are regarded as new hopes for cancer treatment, although the precise assessment of their therapeutic ability needs more studies [5]. Here, we introduce some of the iPSC generated cells applications in cancer immunotherapy.

### 2.1. Using iPSC for Dendritic Cells Generation

Dendritic cells (DCs) are types of hematopoietic cells with potent antigen presenting activity that localize in different tissues of the human body. They have the ability to activate naïve T lymphocytes in an immune response and also have a key role in proliferation of regulatory T-cells or anergy of autoreactive T-cells; they are central components of immune system regulation. When an antigen penetrates into a tissue, site localized DCs capture it by phagocytosis or pinocytosis; peptides that are products of digesting antigens in these cells, then, will be represented to lymphocytes using MHC molecules that can activate them [19].

Because of the large range of antigens that can be presented by DCs, they had been used in most of the cancer immunotherapy as an APC (antigen presenting cell) [20, 21]. Today, it has been revealed that tumor cells express some proteins that are different from normal ones. These antigens are detectable by the immune system but are not sufficient for stimulating an immune response [22]. Then, the basis of using DCs in cancer immunotherapy is presenting sufficient antigens for activating host T-cells against the tumor. In this case, DCs sensitized by tumor cells lysate, synthetic peptides, and complete proteins have been used for stimulation of T-cell response [21, 23, 24].

The first use of DCs for cancer therapy was in 1996 on a patient with follicular B-cell lymphoma. In this study, few numbers of DCs were directly isolated from the patient's blood and underwent spontaneous maturation [25]. In the latter studies, DCs were produced from monocytes isolated from patients peripheral blood [26]. However, this method had some problems such as uneasy proliferation of monocytes* in vitro* [27], limitation in the number of the obtained monocytes, and variable potential of differentiation based on blood donors [13].

In 2000, the first studies on using ESC for DC generation were performed [28]. These ESC-derived DCs could activate a more powerful immune response in comparison to previous studies [20, 28]. However, the unavailability of ESC genetically identical for each patient and the ethical issues in using human ESC create limitations for generating DC from ESC. Both of these problems have been solved using iPS cells [29].

The iPS cell-derived DCs have the characteristics of original DCs including the capability of T-cell stimulation, processing and presenting antigens, and the capability of producing cytokines. While using the OP9 culture system is the main method for generating DCs from iPSC, the xeno-free culture systems also are available to generate iPSC-DCs for clinical use [13, 29]. One of these reports belongs to Choi et al. that generate myelomonocytic cells, including DC, from human iPS cells [30]. Similar results are also indicated in the study of Senju et al. [29] and Zhang et al. [31] on the iPSCs derived from mouse cell lines.

iPS cells can generate hematopoietic cells similar to those derived from ES cells that are specific for each person and can be differentiated from a small number of available somatic cells such as fibroblast, but with a low efficiency [32]. Enhancement of iPSC-derived DCs apoptosis, limitation in cell growth and reduction in colony formation ability of these cells [33], and the problems of cost and time related to iPSC also exist [32]. Because of these limitations, iPSC-derived DCs have not been used in trial studies, yet.

Most of the studies on cancer immunotherapy using DCs have been done for melanoma antigen presentation [9, 20, 34, 35]. The other studied cancers are prostate cancer [36], renal cell carcinoma [37], breast cancer [2, 38], hepatocellular carcinoma [39], multiple myeloma [40], leukemia [20], colorectal cancer [41], gastric cancer [42], and glioblastoma [22, 43]. Cells used in these researches for DC generation were mature and immature monocytes, CD34+ progenitors, ESC, and iPSC, while most of the trial studies were performed using mature monocyte-derived DCs and also CD34+ progenitors-derived DCs that differentiated using cytokines such as TNF-*α*, GM-CSF, and CD40L [9, 11, 34, 35]. These factors in addition to PGE2, IL-6, IL-12, IL-15, and IFN-*γ* were also used for stimulating differentiated DC [20, 40]. Some of the antigens that successfully have been presented by DC cells in these studies include oncogenes (such as RAS), epidermal growth factor receptor (HER-2/neu), embryonic genes (such as MAGE, BAGE, and GACE), normal development genes (such as tyrosinase, gp100, and MART-1/Melan-A), viral genes (such as HPV), and other tumor-associated proteins (such as PSMA and MUCI) [23].

### 2.2. Using iPS for T-Cell Generation

The principal mechanism of tumor immunity is killing of tumor cells by CD8+ CTLs. CTLs have a critical function by recognizing and killing potentially malignant cells. The malignant cells express peptides derived from mutant cellular proteins or oncogenic viral proteins and present them in association with class I MHC molecules. The activation of tumor-specific T-cells depends on DCs, which endocytose tumor cell debris and apoptotic vesicles. After intracellular processing, DCs present peptides derived from tumor-associated antigens in complex with MHC class I molecules to naive CD8+ T-cells. As soon as effector CTLs are generated, they are able to recognize and kill the tumor cells [44–47].

Then, the CD8+ T-cell response is specific for tumor antigens and requires cross-presentation of the tumor antigens by professional APCs, such as dendritic cells. The APCs express costimulator proteins that provide the signals needed for differentiation of CD8+ T-cells into antitumor CTLs. The APCs also express class II MHC molecules that present internalized tumor antigens and activate CD4+ helper T-cells as well [48].

CD4+ cells play their role in antitumor immune responses by providing cytokines such as interleukin-2 (IL-2) (for effective CTL development and clonal expansion of activated CTLs) [49], TNF, and IFN-*γ* (that can boost cellular components of the innate immunity (macrophages and NK cells), increasing tumor cell class I MHC expression and sensitivity to lysis by CTLs) [50, 51]. Furthermore, activated CD4+ T-cells can enhance the function of DCs to induce CTLs [52, 53]. Another subtype of CD4+ T-cell that is often present in tumor tissue is regulatory T-cell (Treg) that negatively regulates the immune system. It differentiates from CD4+ T-cell when recognizing antigens in a noninflamed condition and in the presence of TGF-beta and IL-10. Existence of Treg cells in tumor tissue can decrease the expansion of CTLs and suppress the antitumor immune responses, so they are considered as targets for cancer immunotherapy [1, 53]. The ability of CTLs to provide effective antitumor immunity* in vivo* is most clearly seen in animal experiments using carcinogen and DNA virus-induced tumors. In addition, researches showed that tumor-infiltrating CD8+ cytotoxic T-cells can predict clinical outcome in colon, lung, and breast cancers [54].

Declared activation of tumor-specific CTLs is the main goal of cancer immunotherapy; so, adoptive transfer of tumor-specific T-cells is one of the effective therapeutic approaches for fighting against many types of malignancies [55–57]. The isolation of tumor-specific T-cells from a cancer patient,* in vitro* preparation (activation and expansion), and transfusion of these T-cells to the patient are basic steps of adaptive immunotherapy with T-cell [55], although there are some problems with this approach, for example, the low number of antigen-specific T-cells and senescence of these activated cells [55, 56, 58]. Then, iPSC technology can be used to improve the efficacy of adoptive cell transfer immunotherapy (ACT). The main idea of using this kind of cell is according to the capability of iPSC generation in patient or disease specific noninvasive manner without ethical concerns. The difficulty of obtaining ESCs or HSCs from cancer patients also makes iPSC cells a good option for cancer ACT compared to ESCs or HSCs [45, 56].

Previous studies showed that HSC and ESC can differentiate into lymphocyte lineage using the* in vitro* OP9 coculture system which included OP9 cells expressing a Notch ligand, delta-like 1 [59, 60]. Lei et al. differentiated mouse iPS-MEF-Ng-20D-17 cell line. The iPSC in this study was obtained from mouse embryonic fibroblasts induced through retroviral transfection of Oct3/4, Sox2, Klf4, and c-Myc (Yamanaka factors) into T-cell lineages by culturing it on monolayer OP9-DL1 cell system in addition to Flt-3 ligand and IL-7. Adaptive transfer of these iPS cell-derived T lymphocytes to Rag1-deficient mice (mice lacking mature T-cells) enabled them to reconstitute T-cell pool by generation of CD4+ and CD8+ T lymphocytes in lymph nodes and spleen [61].

An important advance in iPSC research was successful iPSC generation from reprogrammed primary CD34+ hematopoietic progenitor cells obtained from peripheral blood [62, 63]. However, due to the low number of these progenitor cells in nonmobilized adult peripheral blood, various studies tried to generate iPSC from peripheral blood mononuclear cells (PBMCs) [64, 65]. Molecular analysis of PBMC derived iPSC for T-cell receptor and immunoglobulin showed that they are derivations of cells from T lineage and nonlymphoid lineage [65].

A potentially efficient approach for generating antigen-specific CTLs is to generate iPSC from immune T-cells and, after their expansion, redifferentiate into T-cells. Brown and colleagues indicated that human T lymphocyte can act as cell source for iPSC generation. Peripheral blood mononuclear cells (PBMCs) were separated from whole blood by leukapheresis or venipuncture and then CD3+ T-cells were expanded by stimulation with IL-2 and anti-CD3 antibody. T-cell-derived iPSCs (TiPS) were generated from activated T-cell when exposed to retroviral transduction of the reprogramming factors [64]. These T-iPSCs preserve their original T-cell receptor (TCR) gene rearrangements, so they can be used as an unlimited source of hematopoietic stem cells bearing endogenous tumor-specific TCR gene for cancer ACT therapy. These T-iPS cells may bypass key step in the thymic development sequence by differentiating* in vitro* in a thymus-independent manner [64].

Some studies have demonstrated the successful differentiation of antigen specific T-cells from an iPSC that itself was generated from CTL specific for particular epitope [57, 66]. CTLs were transduced with Sendai virus bearing Yamanaka factors (Klf4, Sox2, Oct4, c-Myc, and miR-302 target sequence) and SV40 (large T antigen). Experiments on iPSCs generation from mature CTLs specific for the MART-1 (melanoma epitope) [57] and pp65 antigen (cytomegalovirus) [66] indicate that iPSC-derived CTLs (iPSC-CTLs) retain their original antigen specificity. Stimulation of CTL-iPSC-CTL cells with their specific antigens led to IFN-*γ* secretion and degranulation in a normal manner represents their normal and specific cytolytic reactivity [57, 66]. CTL-iPSC-CTL cells have some differences to parent CD8+ T-cells with elongated telomeres and excellent potential for proliferation and survival. Additionally, some of them display central memory T-cell (T_CM_) and stem-cell memory T-cells (T_SCM_) phenotypes which was associated with increasing expression of CCR7, CD27, and CD28 markers [57, 58, 66]. Several lines of evidence show that T_SCM_ and T_CM_ have superior antitumor immunity for ACT-based immunotherapy (due to the resistance to apoptosis, potent response to homeostatic cytokines, self-renewal, and efficient generation of other T-cells' population) [66–72].

Also, generation of iPSCs from murine splenic B-cell and redifferentiation into T-cell lineage have been reported. Isolated B-cells (CD19+, CD24+, and CD45R+ (B220+) and IgM+) were activated with IL-4 and LPS and then transduced with four retroviruses encoding reprogramming factors. Using OP9 coculture system, these B-iPS cells have been differentiated to T-cells that keep their original BCR rearrangement. iPS cell-derived T-cells contained both CD4/CD8 double-positive and CD8+ cells that have surface expression of TCR*αβ* and TCR*γδ* with normal function following TCR stimulation [73]. Further studies indicated that B-cell-derived iPSCs (B-iPS) and T-cell-derived iPSCs (TiPS) have the same characteristics as human embryonic stem cells (hESCs) [64, 73].

Combination of iPS generation technology with transduction of tumor antigen-specific T-cell receptors (TCRs) or chimeric antigen receptors (CARs) showed successful generation of tumor-specific transgene T-cells. This approach can solve the problems related to low number of tumor-specific T-cells in peripheral blood of patients, their recognition and separation, and invasive nature of biopsy [45, 46, 74]. Lei and colleagues used murine iPS cells for introducing a retrovirus vector encoding MHCI restricted ovalbumin- (OVA-) specific TCR (OT-I). OT-I/iPS cells developed to CD8 CTL following adoptive transfer into recipient mice, produced IL-2 and interferon (IFN) after stimulation, and penetrated into tumor tissue after adoptive transfer [45, 46]. This study showed that number of specific CTLs increased in lymph node and spleen after ACT in mice [46]. Also, cells could infiltrate into tumor tissue and 90-fold greater target cell lysis has been seen in these mice compared to control mice [45, 46]. Comparison of survival after ACT in two groups of tumor bearing mice receiving TCR gene-transduced iPS cells showed 100% survival of iPSCs receiving mice in comparison to CD8+ T-cells receiving mice [46].

**Figure 2:**
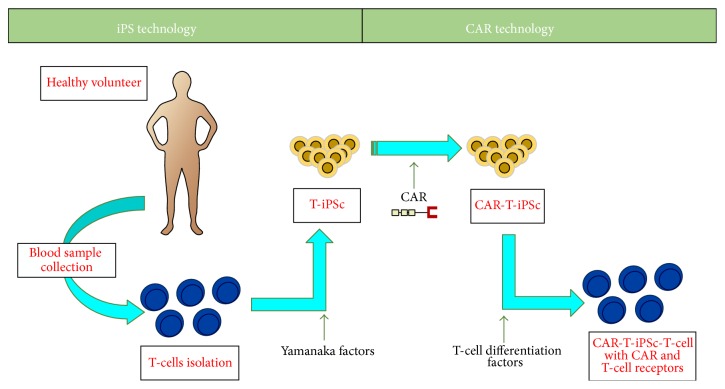
Differentiation of 1928z CAR engineered T-iPSCs into CD19-specific functional T lymphocytes.

In just one study, genetic modification was performed using the CAR technology. T-iPSCs are generated by retrovirus reprogrammed T-cells isolated from peripheral blood of healthy volunteers. In the next step, CAR sequence specific for CD19 has been added to human T-iPSCs colonies (Figure 2). iPSC-derived CAR specific T-cells were phenotypically similar to innate T*γδ* cells and after ACT to mice showed potential ability to inhibit tumor progression in xenograft model [74]. Although the combination of iPS and TCRs/CARs techniques is efficient and may remove necessity for the detection of antigen-specific T-cells, this approach is costly and there are insertional mutagenesis risks [56].

OP-9 coculture system was not able to generate iPSCs-CD4+ T-cells* in vitro* [57, 61, 66, 73] because of the limitation in MHCII expression by OP9 cells [75, 76]. Normal development of CD4+ CD8 T-cells occurs by interaction with MHCII of thymic epithelium and expression of ThPOK, TOX, GATA-3, and RUNX factors essential for CD4+ lineage generation* in vivo* [61]. Only one study has shown the detection of few mature CD4+ T-cells in culture [16]. According to importance of CD4+ helper T-cells in antitumor immunity by promoting the permanence of memory CD8 T-cells [53], isolation of regulatory T-cells (Treg) based on CD4+ CD25+ CD127^low^ CD45RA+ from tumor microenvironment and reprogramming them into iPSCs and then generation of CD4+ helper T-cells may be an effective strategy for ACT [56].

Despite the great advantages of ACT with iPSC-derived T-cells in cancerous mice model [45, 46, 74], there are some limitations when applied* in vivo*; for example, differentiation of iPSC-derived T-cells takes a long time (at least six weeks) and because of their origin there is a teratoma genesis risk [45, 46]. However, the risk of tumorigenesis of iPSC-derived T-cells is just reported in one study. In the study of Lei and colleagues, when they used* in vivo* induction system for generation of antigen-specific T-cell differentiation from iPS cells, they did not observe any extrathymic mass in C57BL/6 mice although they observed an extrathymic mass in only one of the Rag1−/− mice. This finding can clear the importance of iPS genetic background for* in vivo* differentiation [45]. Other limitations are osteoporosis, hair loss, and autoimmune manifestation without any clear reasons (one explanation for it may be* in vivo* differentiation of other immune cells from iPSCs) [45, 66].

The immunogenicity of iPSC-derived cells is very complicated. Zhao et al. found that some but not all cells derived from mouse iPSC can be immunogenic and this immune rejection response was T-cell dependent. They reported that the inbred C57BL/6 (B6) iPSCs and their derived teratomas can induce T-cell-dependent immune responses after transplantation into the syngeneic B6 mice, although based on their report the immunogenicity of iPS was lower than ESC* in vivo* [77]. Abe's group studied the immunogenicity of different cell types derived from iPSCs including skin cells, bone marrow cells, and cardiomyocytes. This study indicated that iPSC-derived cardiomyocytes are highly immunogenic, although iPSC-derived skin and bone marrow cells have lower immunogenic effect [78].

It is widely accepted that reprogramming process can induce both genetic and epigenetic defects in produced iPSCs [79–82]. Abnormal overexpression of Hormad1, Zg16, Cyp3a11, Lce1f, Spt1, Lce3a, Chi3L4, Olr1, and Retn genes was also shown in iPSC-derived cells by gene expression analysis. These genes can be effective in immunogenicity and stimulation of T-cell-mediated immune response after ACT [77]; so, the assessment of iPSC-derived T-cells immunogenicity should be considered before their clinical applications for cancer immunotherapy [45].

Researches showed that changing culture condition and adding multiple soluble proteins influence iPSC lineage differentiation. Presence of transforming growth factor-*β* (TGF-*β*) along with TCR stimulation led to differentiation of suppressor T-cells (Foxp3+ population) in iPSC-derived T-cells culture [73]. Also, it has been shown that stimulation of T-cells in the presence of IL-7, IL-15, and IL-21 results in memory phenotype with enhanced persistence in comparison to IL-2 primed T-cells before ACT [83–87] and inhibition of GSK3b (glycogen synthase kinase 3 beta) led to more efficient production of TSCM population* in vitro* [66]. So, combination of iPSC technology with CAR/TCR transgene technique [88] and optimization of culture media [66] may improve the iPSC-derived T-cells that are suitable for clinical applications in cancer ACT.

### 2.3. Using iPS for Cytokine Producing Cell Generation

One of the mechanisms used by tumor cells for escaping immune response is the suppression of immune response in the tumor area with secretion of immune suppressor cytokines [7] (such as PGE2, IDO, and TGF-*β* [14]). Then, the basis of using cytokine producing cells in cancers is generating cells with the capability of migration into tumor tissue and secretion of cytokines with the immune activation ability in this area [14–16, 89, 90]. Cytokine producing cells can be from myeloid or lymphoid lineages obtained from coculturing of iPSC or ESC with a mouse bone marrow stromal cell line (OP9) [16] and using of different growth factor (regarding cell type that it must be differentiated into).

One of the most effective cells in defense against tumor development with cytokine secretion activity is natural killer (NK) cells. NK cells have been used in some clinical studies including AML and some other hematological malignancies with low toxicity for patients [14]. However, a significant factor in success of treatment is obtaining a pure and functional NK cell population [91]. NK cell has been differentiated from both ESC and iPSC using two different sets of factors (IL-15, IL-3, IL-7, Flt-3L, SCF or BMP4, VEGF, SCF, FGF, TPO, and Flt-3L). These cells were reported to have the ability to secrete cytokines such as IFN-*γ*, in addition to their ability for cell-mediated toxicity [92].

The other cytokine producing cell is T lymphocyte that has been reported to have the ability to produce cytokines such as TNF-*α*, IFN-*γ*, and IL-2 and cytolytic proteins (perforin and granzyme B) when differentiated from iPSC [16]. T-cells with the ability to secrete CSF also have been reported to be effective in patients with myelodysplastic syndromes [93].

Macrophages are cells whose infiltration is frequently observed in cancer area [94]. This kind of cell has two different functions related to cancer: (1) tumor-associated macrophages (TAM) that cause cancer progression and (2) other macrophages with antitumor activity. Cancer immunotherapy using macrophages just like using NK cells has great dependence on taking the efficient number of cells; this large number of macrophages is achievable by using ESC or iPSC for differentiation into it [15].

Genetically modified iPSCs that differentiated into myeloid lineage (iPS-ML) with the capability of cytokine secretion are also reported to be effective in cancer cells elimination. iPS-ML cells producing IFN-*α*, IFN-*β*, and IFN-*γ* and TNF-*α* have been studied in gastric cancer cell line (NUGC-4) and human pancreatic cell line (MIAPaCa-2). In this study also, the production of anti-HER2 antibody (in ScFV form) using iPS-ML cells has been reported. The iPS-ML cells had the ability to infiltrate into tumor area and produce antibody and cytokines in the tumor site. The result of this study also indicated that IFN-*β* has greater local concentration and remains longer than IFN-*α* in cancer tissue in the SCID mice model [15].

## 3. Conclusion

iPS cells have been studied in different fields of cancer immunotherapy including tumor Ag presentation, T-cell activity regulation, and cytokine or Ab producing cells, many of which had a successful result for elimination of cancer cell lines. There are many hopes for the future of this technique, although it cannot be used in clinical treatment because of some obstacles that already exist such as generating hiPSC (human iPSC) in a safe manner, enhancing reprogramming and differentiation process efficiency [14], reducing the time and cost needed for the process [13], and proving iPSC safety for clinical use. These problems must be solved before any use of iPSC in patients treatment [14].

Taken together, cancer immunotherapy with iPSC can be considered as a new hope for cancer treatment but still on the early stages that need more studies before its real use in clinic [40].

## References

[1] Raval R. R., Sharabi A. B., Walker A. J., Drake C. G., Sharma P. (2014). Tumor immunology and cancer immunotherapy: summary of the 2013 SITC primer. *Journal for ImmunoTherapy of Cancer*.

[2] Koski G. K., Koldovsky U., Xu S. (2012). A novel dendritic cell-based immunization approach for the induction of durable Th1-polarized Anti-HER-2/neu responses in women with early breast cancer. *Journal of Immunotherapy*.

[3] Meissner M., Reichert T. E., Kunkel M. (2005). Defects in the human leukocyte antigen class I antigen-processing machinery in head and neck squamous cell carcinoma: association with clinical outcome. *Clinical Cancer Research*.

[4] Medema J. P., De Jong J., Peltenburg L. T. C. (2001). Blockade of the granzyme B/perforin pathway through overexpression of the serine protease inhibitor PI-9/SPI-6 constitutes a mechanism for immune escape by tumors. *Proceedings of the National Academy of Sciences of the United States of America*.

[5] Töpfer K., Kempe S., Müller N. (2011). Tumor evasion from T cell surveillance. *Journal of Biomedicine and Biotechnology*.

[6] Bogdahn U., Hau P., Stockhammer G. (2011). Targeted therapy for high-grade glioma with the TGF-*β*2 inhibitor trabedersen: results of a randomized and controlled phase IIb study. *Neuro-Oncology*.

[7] Kerkar S. P., Restifo N. P. (2012). Cellular constituents of immune escape within the tumor microenvironment. *Cancer Research*.

[8] Dietrich K., Theobald M. (2015). Immunologische tumor therapie. *Der Internist*.

[9] Chiang C. L.-L., Maier D. A., Kandalaft L. E. (2011). Optimizing parameters for clinical-scale production of high IL-12 secreting dendritic cells pulsed with oxidized whole tumor cell lysate. *Journal of Translational Medicine*.

[10] Korman A. J., Peggs K. S., Allison J. P. (2006). Checkpoint blockade in cancer immunotherapy. *Advances in Immunology*.

[11] Senju S., Haruta M., Matsunaga Y. (2009). Characterization of dendritic cells and macrophages generated by directed differentiation from mouse induced pluripotent stem cells. *STEM CELLS*.

[12] Patel M., Yang S. (2010). Advances in reprogramming somatic cells to induced pluripotent stem cells. *Stem Cell Reviews and Reports*.

[13] Senju S., Matsunaga Y., Fukushima S. (2011). Immunotherapy with pluripotent stem cell-derived dendritic cells. *Seminars in Immunopathology*.

[14] Eguizabal C., Zenarruzabeitia O., Monge J. (2014). Natural killer cells for cancer immunotherapy: pluripotent stem cells-derived NK cells as an immunotherapeutic perspective. *Frontiers in Immunology*.

[15] Koba C., Haruta M., Matsunaga Y. (2013). Therapeutic effect of human iPS-cell-derived myeloid cells expressing IFN-*β* against peritoneally disseminated cancer in xenograft models. *PLoS ONE*.

[16] Chang C.-W., Lai Y.-S., Lamb L. S., Townes T. M. (2014). Broad T-cell receptor repertoire in T-lymphocytes derived from human induced pluripotent stem cells. *PLoS ONE*.

[17] Morishima T., Watanabe K., Niwa A. (2011). Neutrophil differentiation from human-induced pluripotent stem cells. *Journal of Cellular Physiology*.

[18] Haruta M., Tomita Y., Yuno A. (2013). TAP-deficient human iPS cell-derived myeloid cell lines as unlimited cell source for dendritic cell-like antigen-presenting cells. *Gene Therapy*.

[19] Antignano F., Ibaraki M., Kim C. (2010). SHIP is required for dendritic cell maturation. *Journal of Immunology*.

[20] Ballestrero A., Boy D., Moran E., Cirmena G., Brossart P., Nencioni A. (2008). Immunotherapy with dendritic cells for cancer. *Advanced Drug Delivery Reviews*.

[21] Su Z., Frye C., Bae K.-M., Kelley V., Vieweg J. (2008). Differentiation of human embryonic stem cells into immunostimulatory dendritic cells under feeder-free culture conditions. *Clinical Cancer Research*.

[22] Liau L. M., Black K. L., Martin N. A. (2000). Treatment of a patient by vaccination with autologous dendritic cells pulsed with allogeneic major histocompatibility complex class I-matched tumor peptides. Case Report. *Neurosurgical Focus*.

[23] Tewari M., Sahai S., Mishra R. R., Shukla S. K., Shukla H. S. (2012). Dendritic cell therapy in advanced gastric cancer: a promising new hope?. *Surgical Oncology*.

[24] Iwamoto H., Ojima T., Nakamori M. (2013). Cancer vaccine therapy using genetically modified induced pluripotent stem cell-derived dendritic cells expressing the TAA gene. *Gan to Kagaku Ryōhōsha*.

[25] Hsu F. J., Benike C., Fagnoni F. (1996). Vaccination of patients with B-cell lymphoma using autologous antigen-pulsed dendritic cells. *Nature Medicine*.

[26] Jakobsen M. A., Møller B. K., Lillevang S. T. (2004). Serum concentration of the growth medium markedly affects monocyte-derived dendritic cells' phenotype, cytokine production profile and capacities to stimulate in MLR. *Scandinavian Journal of Immunology*.

[27] Matsunaga Y., Fukuma D., Hirata S. (2008). Activation of antigen-specific cytotoxic T lymphocytes by *β*
_2_-microglobulin or TAP1 gene disruption and the introduction of recipient-matched MHC class I gene in allogeneic embryonic stem cell-derived dendritic cells. *The Journal of Immunology*.

[28] Fairchild P. J., Brook F. A., Gardner R. L. (2000). Directed differentiation of dendritic cells from mouse embryonic stem cells. *Current Biology*.

[29] Senju S., Haruta M., Matsumura K. (2011). Generation of dendritic cells and macrophages from human induced pluripotent stem cells aiming at cell therapy. *Gene Therapy*.

[30] Choi K.-D., Vodyanik M. A., Slukvin I. I. (2009). Generation of mature human myelomonocytic cells through expansion and differentiation of pluripotent stem cell–derived lin^–^CD34^+^CD43^+^CD45^+^ progenitors. *The Journal of Clinical Investigation*.

[31] Zhang Q., Fujino M., Iwasaki S. (2014). Generation and characterization of regulatory dendritic cells derived from murine induced pluripotent stem cells. *Scientific Reports*.

[32] Senju S., Hirata S., Motomura Y. (2010). Pluripotent stem cells as source of dendritic cells for immune therapy. *International Journal of Hematology*.

[33] Huang G. T.-J. (2010). Induced pluripotent stem cells—a new foundation in medicine. *Journal of Experimental and Clinical Medicine*.

[34] Thurner B., Haendle I., Röder C. (1999). Vaccination with Mage-3A1 peptide-pulsed nature, monocyte-derived dendritic cells expands specific cytotoxic T cells and induces regression of some metastases in advanced stage IV melanoma. *The Journal of Experimental Medicine*.

[35] Banchereau J., Palucka A. K., Dhodapkar M. (2001). Immune and clinical responses in patients with metastatic melanoma to CD34^+^ progenitor-derived dendritic cell vaccine. *Cancer Research*.

[36] Fong L., Brockstedt D., Benike C. (2001). Dendritic cell-based xenoantigen vaccination for prostate cancer Immunotherapy. *Journal of Immunology*.

[37] Höltl L., Zelle-Rieser C., Gander H. (2002). Immunotherapy of metastatic renal cell carcinoma with tumor lysate-pulsed autologous dendritic cells. *Clinical Cancer Research*.

[38] Baek S., Kim C.-S., Kim S.-B. (2011). Combination therapy of renal cell carcinoma or breast cancer patients with dendritic cell vaccine and IL-2: results from a phase I/II trial. *Journal of Translational Medicine*.

[39] Motomura Y., Senju S. S., Nakatsura T. (2006). Embryonic stem cell-derived dendritic cells expressing glypican-3, a recently identified oncofetal antigen, induce protective immunity against highly metastatic mouse melanoma, B16-F10. *Cancer Research*.

[40] Schuler G., Schuler-Thurner B., Steinman R. M. (2003). The use of dendritic cells in cancer immunotherapy. *Current Opinion in Immunology*.

[41] Bao C.-Q., Jin C., Xu B.-H., Gu Y.-L., Li J.-P., Xiao-Lu (2011). Vaccination with apoptosis colorectal cancer cell pulsed autologous dendritic cells in advanced colorectal cancer patients: report from a clinical observation. *African Journal of Biotechnology*.

[42] Ananiev J., Gulubova M. V., Manolova I. (2011). Prognostic significance of CD83 positive tumor-infiltrating dendritic cells and expression of TGF-beta 1 in human gastric cancer. *Hepato-Gastroenterology*.

[43] Nakano I., Chiocca E. A. (2012). Hope and challenges for dendritic cell-based vaccine therapy for glioblastoma. *World Neurosurgery*.

[44] Kayagaki N., Yamaguchi N., Nakayama M., Hiroshi E., Okumura K., Yagita H. (1999). Type I interferons (IFNs) regulate tumor necrosis factor-related apoptosis-inducing ligand (TRAIL) expression on human T cells: a novel mechanism for the antitumor effects of type I IFNs. *Journal of Experimental Medicine*.

[45] Lei F., Haque R., Xiong X., Song J. (2012). Directed differentiation of induced pluripotent stem cells towards T lymphocytes. *Journal of Visualized Experiments*.

[46] Lei F., Zhao B., Haque R. (2011). In vivo programming of tumor antigen-specific T lymphocytes from pluripotent stem cells to promote cancer immunosurveillance. *Cancer Research*.

[47] Rousalova I., Krepela E. (2010). Granzyme B-induced apoptosis in cancer cells and its regulation (review). *International Journal of Oncology*.

[48] Platzer B., Stout M., Fiebiger E. (2014). Antigen cross-presentation of immune complexes. *Frontiers in Immunology*.

[49] Clarke S. R. (2000). The critical role of CD40/CD40L in the CD4-dependent generation of CD8+ T cell immunity. *Journal of Leukocyte Biology*.

[50] Le Page C., Génin P., Baines M. G., Hiscott J. (2000). Interferon activation and innate immunity. *Reviews in Immunogenetics*.

[51] Seliger B., Ruiz-Cabello F., Garrido F. (2008). IFN inducibility of major histocompatibility antigens in tumors. *Advances in Cancer Research*.

[52] Cella M., Scheidegger D., Palmer-Lehmann K., Lane P., Lanzavecchia A., Alber G. (1996). Ligation of CD40 on dendritic cells triggers production of high levels of interleukin-12 and enhances T cell stimulatory capacity: T-T help via APC activation. *The Journal of Experimental Medicine*.

[53] Kirkwood J. M., Butterfield L. H., Tarhini A. A., Zarour H., Kalinski P., Ferrone S. (2012). Immunotherapy of cancer in 2012. *CA Cancer Journal for Clinicians*.

[54] Corthay A. (2014). Does the immune system naturally protect against cancer?. *Frontiers in Immunology*.

[55] Fernandez T. D. S., de Souza Fernandez C., Mencalha A. L. (2013). Human induced pluripotent stem cells from basic research to potential clinical applications in cancer. *BioMed Research International*.

[56] Sachamitr P., Hackett S., Fairchild P. J. (2014). Induced pluripotent stem cells: challenges and opportunities for cancer immunotherapy. *Frontiers in Immunology*.

[57] Vizcardo R., Masuda K., Yamada D. (2013). Regeneration of human tumor antigen-specific T cells from iPSCs derived from mature CD8^+^ T cells. *Cell Stem Cell*.

[58] Crompton J. G., Rao M., Restifo N. P. (2013). Memoirs of a reincarnated T cell. *Cell Stem Cell*.

[59] La Motte-Mohs R. N., Herer E., Zúňiga-Pflücker J. C. (2005). Induction of T-cell development from human cord blood hematopoietic stem cells by Delta-like 1 in vitro. *Blood*.

[60] Schmitt T. M., de Pooter R. F., Gronski M. A., Cho S. K., Ohashi P. S., Zúñiga-Pflücker J. C. (2004). Induction of T cell development and establishment of T cell competence from embryonic stem cells differentiated in vitro. *Nature Immunology*.

[61] Lei F., Haque R., Weiler L., Vrana K. E., Song J. (2009). T lineage differentiation from induced pluripotent stem cells. *Cellular Immunology*.

[62] Loh Y.-H., Agarwal S., Park I.-H. (2009). Generation of induced pluripotent stem cells from human blood. *Blood*.

[63] Ye Z., Zhan H., Mali P. (2009). Human-induced pluripotent stem cells from blood cells of healthy donors and patients with acquired blood disorders. *Blood*.

[64] Brown M. E., Rondon E., Rajesh D. (2010). Derivation of induced pluripotent stem cells from human peripheral blood T lymphocytes. *PLoS ONE*.

[65] Loh Y.-H., Hartung O., Li H. (2010). Reprogramming of T cells from human peripheral blood. *Cell Stem Cell*.

[66] Nishimura T., Kaneko S., Kawana-Tachikawa A. (2013). Generation of rejuvenated antigen-specific T cells by reprogramming to pluripotency and redifferentiation. *Cell Stem Cell*.

[67] Hataye J., Moon J. J., Khoruts A., Reilly C., Jenkins M. K. (2006). Naïve and memory CD4^+^ T cell survival controlled by clonal abundance. *Science*.

[68] Hinrichs C. S., Borman Z. A., Gattinoni L. (2011). Human effector CD8^+^ T cells derived from naive rather than memory subsets possess superior traits for adoptive immunotherapy. *Blood*.

[69] Seki Y.-I., Yang J., Okamoto M. (2007). IL-7/STAT5 cytokine signaling pathway is essential but insufficient for maintenance of naive CD4 T cell survival in peripheral lymphoid organs. *Journal of Immunology*.

[70] Siewert C., Lauer U., Cording S. (2008). Experience-driven development: effector/memory-like *α*E^+^Foxp3^+^ regulatory T cells originate from both naive T cells and naturally occurring naive-like regulatory T cells. *Journal of Immunology*.

[71] Stemberger C., Huster K. M., Koffler M. (2007). A single naive CD8^+^ T cell precursor can develop into diverse effector and memory subsets. *Immunity*.

[72] Wang L.-X., Plautz G. E. (2010). Tumor-primed, in vitro-activated CD4^+^ effector T cells establish long-term memory without exogenous cytokine support or ongoing antigen exposure. *The Journal of Immunology*.

[73] Wada H., Kojo S., Kusama C. (2011). Successful differentiation to T cells, but unsuccessful B-cell generation, from B-cell-derived induced pluripotent stem cells. *International Immunology*.

[74] Themeli M., Kloss C. C., Ciriello G. (2013). Generation of tumor-targeted human T lymphocytes from induced pluripotent stem cells for cancer therapy. *Nature Biotechnology*.

[75] Ciofani M., Knowles G. C., Wiest D. L., von Boehmer H., Zúñiga-Pflücker J. C. (2006). Stage-specific and differential notch dependency at the *αβ* and *γδ* T lineage bifurcation. *Immunity*.

[76] Schmitt T. M., Ciofani M., Petrie H. T., Zúñiga-Pflücker J. C. (2004). Maintenance of T cell specification and differentiation requires recurrent notch receptor-ligand interactions. *Journal of Experimental Medicine*.

[77] Zhao T., Zhang Z.-N., Rong Z., Xu Y. (2011). Immunogenicity of induced pluripotent stem cells. *Nature*.

[78] Araki R., Uda M., Hoki Y. (2013). Negligible immunogenicity of terminally differentiated cells derived from induced pluripotent or embryonic stem cells. *Nature*.

[79] Kim K., Doi A., Wen B. (2010). Epigenetic memory in induced pluripotent stem cells. *Nature*.

[80] Doi A., Park I.-H., Wen B. (2009). Differential methylation of tissue- and cancer-specific CpG island shores distinguishes human induced pluripotent stem cells, embryonic stem cells and fibroblasts. *Nature Genetics*.

[81] Polo J. M., Liu S., Figueroa M. E. (2010). Cell type of origin influences the molecular and functional properties of mouse induced pluripotent stem cells. *Nature Biotechnology*.

[82] Lister R., Pelizzola M., Kida Y. S. (2011). Hotspots of aberrant epigenomic reprogramming in human induced pluripotent stem cells. *Nature*.

[83] Hinrichs C. S., Spolski R., Paulos C. M. (2008). IL-2 and IL-21 confer opposing differentiation programs to CD8^+^ T cells for adoptive immunotherapy. *Blood*.

[84] Moroz A., Eppolito C., Li Q., Tao J., Clegg C. H., Shrikant P. A. (2004). IL-21 enhances and sustains CD8^+^ T cell responses to achieve durable tumor immunity: comparative evaluation of IL-2, IL-15, and IL-21. *The Journal of Immunology*.

[85] Neeson P., Shin A., Tainton K. M. (2010). Ex vivo culture of chimeric antigen receptor T cells generates functional CD8^+^ T cells with effector and central memory-like phenotype. *Gene Therapy*.

[86] Schluns K. S., Lefrançois L. (2003). Cytokine control of memory T-cell development and survival. *Nature Reviews Immunology*.

[87] Zeng R., Spolski R., Finkelstein S. E. (2005). Synergy of IL-21 and IL-15 in regulating CD8^+^ T cell expansion and function. *The Journal of Experimental Medicine*.

[88] Singh H., Figliola M. J., Dawson M. J. (2011). Reprogramming CD19-specific T cells with IL-21 signaling can improve adoptive immunotherapy of B-lineage malignancies. *Cancer Research*.

[89] Zhang L., Song X., Mohri Y., Qiao L. (2015). Role of inflammation and tumor microenvironment in the development of gastrointestinal cancers: what induced pluripotent stem cells can do?. *Current Stem Cell Research & Therapy*.

[90] Haga E., Endo Y., Haruta M. (2014). Therapy of peritoneally disseminated colon cancer by TAP-Deficient embryonic stem cell-derived macrophages in allogeneic recipients. *Journal of Immunology*.

[91] Bock A. M., Knorr D., Kaufman D. S. (2013). Development, expansion, and in vivo monitoring of human NK cells from human embryonic stem cells (hESCs) and and induced pluripotent stem cells (iPSCs). *Journal of Visualized Experiments*.

[92] Larbi A., Gombert J.-M., Auvray C. (2012). The HOXB4 homeoprotein promotes the ex vivo enrichment of functional human embryonic stem cell-derived NK cells. *PLoS ONE*.

[93] Merchav S., Nagler A., Sahar E., Tatarsky I. (1987). Production of human pluripotent progenitor cell colony stimulating activity (CFU-GEMMCSA) in patients with myelodysplastic syndromes. *Leukemia Research*.

[94] Lewis C. E., Pollard J. W. (2006). Distinct role of macrophages in different tumor microenvironments. *Cancer Research*.

